# An overview of the medical-physics-related verification system for radiotherapy multicenter clinical trials by the Medical Physics Working Group in the Japan Clinical Oncology Group–Radiation Therapy Study Group

**DOI:** 10.1093/jrr/rraa089

**Published:** 2020-09-29

**Authors:** Teiji Nishio, Mitsuhiro Nakamura, Hiroyuki Okamoto, Satoshi Kito, Toshiyuki Minemura, Shuichi Ozawa, Yu Kumazaki, Masayori Ishikawa, Naoki Tohyama, Masahiko Kurooka, Takeo Nakashima, Hidetoshi Shimizu, Ryusuke Suzuki, Satoshi Ishikura, Yasumasa Nishimura

**Affiliations:** Department of Medical Physics, Graduate School of Medicine, Tokyo Women’s Medical University, 8-1, Kawada-cho, Shinjuku-ku, Tokyo 162-8666, Japan; Medical Physics Working Group (MPWG) in Japan Clinical Oncology Group - Radiation Therapy Study Group (JCOG-RTSG), Tokyo, Japan; Division of Medical Physics, Department of Information Technology and Medical Engineering, Human He Sciences, Graduate School of Medicine, Kyoto University, 53 Shogoin-Kawaharacho, Sakyo-ku, Kyoto 606-8507, Japan; Medical Physics Working Group (MPWG) in Japan Clinical Oncology Group - Radiation Therapy Study Group (JCOG-RTSG), Tokyo, Japan; Department of Medical Physics, National Cancer Center Hospital, 5-1-1 Tsukiji, Chuo-ku, Tokyo, 104-0045 Japan; Medical Physics Working Group (MPWG) in Japan Clinical Oncology Group - Radiation Therapy Study Group (JCOG-RTSG), Tokyo, Japan; Department of Radiology, Tokyo Metropolitan Bokutoh Hospital, 4-23-15 Kotobashi, Sumida-ku, Tokyo 130-8575, Japan; Department of Radiation Oncology, Tokyo Metropolitan Cancer and Infectious Disease Center Komagome Hospital, 3-18-22 Honkomagome, Bunkyo-ku, Tokyo 113-8677, Japan; Division of Medical Physics, Department of Information Technology and Medical Engineering, Human He Sciences, Graduate School of Medicine, Kyoto University, 53 Shogoin-Kawaharacho, Sakyo-ku, Kyoto 606-8507, Japan; Medical Physics Working Group (MPWG) in Japan Clinical Oncology Group - Radiation Therapy Study Group (JCOG-RTSG), Tokyo, Japan; Division of Medical Support and Partnership, Center for Cancer Control and Information Services, National Cancer Center, 5-1-1 Tsukiji, Chuo-ku, Tokyo 104-0045, Japan; Medical Physics Working Group (MPWG) in Japan Clinical Oncology Group - Radiation Therapy Study Group (JCOG-RTSG), Tokyo, Japan; Department of Radiation Oncology, Graduate School of Biomedical and Health Sciences, Hiroshima University, 1-2-3 Kasumi, Minami-ku, Hiroshima 734-8551, Japan; Hiroshima High-Precision Radiotherapy Cancer Center, 3-2-2, Futabanosato, Higashi-ku, Hiroshima 732-0057, Japan; Medical Physics Working Group (MPWG) in Japan Clinical Oncology Group - Radiation Therapy Study Group (JCOG-RTSG), Tokyo, Japan; Department of Radiation Oncology, Saitama Medical University International Medical Center, 1397-1 Yamane, Hidaka, Saitama 350-1298, Japan; Medical Physics Working Group (MPWG) in Japan Clinical Oncology Group - Radiation Therapy Study Group (JCOG-RTSG), Tokyo, Japan; Faculty of Health Sciences, Hokkaido University, N-12 W-5 Kita-ku, Sapporo, 060-0812, Japan; Medical Physics Working Group (MPWG) in Japan Clinical Oncology Group - Radiation Therapy Study Group (JCOG-RTSG), Tokyo, Japan; Division of Medical Physics, Tokyo Bay Advanced Imaging & Radiation Oncology Makuhari Clinic, 1-17 Toyosuna, Mihama-ku, Chiba, 261-0024, Japan; Medical Physics Working Group (MPWG) in Japan Clinical Oncology Group - Radiation Therapy Study Group (JCOG-RTSG), Tokyo, Japan; Department of Radiation Therapy, Tokyo Medical University Hospital, 6-7-1, Nishishinjuku, Shinjuku-ku, Tokyo 160-0023, Japan; Medical Physics Working Group (MPWG) in Japan Clinical Oncology Group - Radiation Therapy Study Group (JCOG-RTSG), Tokyo, Japan; Radiation Therapy Section, Department of Clinical Support, Hiroshima University Hospital, 1-2-3 Kasumi, Minami-ku, Hiroshima 734-8551, Japan; Medical Physics Working Group (MPWG) in Japan Clinical Oncology Group - Radiation Therapy Study Group (JCOG-RTSG), Tokyo, Japan; Department of Radiation Oncology, Aichi Cancer Center Hospital, 1-1 Kanokoden, Chikusa-ku, Nagoya, Aichi 464-8681, Japan; Medical Physics Working Group (MPWG) in Japan Clinical Oncology Group - Radiation Therapy Study Group (JCOG-RTSG), Tokyo, Japan; Department of Medical Physics, Hokkaido University Hospital, North-14, West-5, Kita-Ku, Sapporo, Hokkaido 060-8638, Japan; Medical Physics Working Group (MPWG) in Japan Clinical Oncology Group - Radiation Therapy Study Group (JCOG-RTSG), Tokyo, Japan; Department of Radiology, Graduate School of Medical Sciences, Nagoya City University, 1 Kawasumi, Mizuho-cho, Mizuho-ku, Nagoya, Aichi 467-8601, Japan; Radiotherapy Committee (RC) in Japan Clinical Oncology Group, Tokyo, Japan; Japan Clinical Oncology Group - Radiation Therapy Study Group (JCOG-RTSG), Tokyo, Japan; Department of Radiation Oncology, Kindai University Faculty of Medicine, 377-2 Ohno-Higashi, Osaka-Sayama, Osaka 589-8511, Japan; Japan Clinical Oncology Group - Radiation Therapy Study Group (JCOG-RTSG), Tokyo, Japan

**Keywords:** dosimetry audit, irradiation localization audit, credentialing, radiotherapy clinical trial

## Abstract

The Japan Clinical Oncology Group*–*Radiation Therapy Study Group (JCOG-RTSG) has initiated several multicenter clinical trials for high-precision radiotherapy, which are presently ongoing. When conducting multi-center clinical trials, a large difference in physical quantities, such as the absolute doses to the target and the organ at risk, as well as the irradiation localization accuracy, affects the treatment outcome. Therefore, the differences in the various physical quantities used in different institutions must be within an acceptable range for conducting multicenter clinical trials, and this must be verified with medical physics consideration. In 2011, Japan’s first Medical Physics Working Group (MPWG) in the JCOG-RTSG was established to perform this medical-physics-related verification for multicenter clinical trials. We have developed an auditing method to verify the accuracy of the absolute dose and the irradiation localization. Subsequently, we credentialed the participating institutions in the JCOG multicenter clinical trials that were using stereotactic body radiotherapy (SBRT) for lungs, intensity-modulated radiotherapy (IMRT) and volumetric-modulated arc therapy (VMAT) for several disease sites, and proton beam therapy (PT) for the liver. From the verification results, accuracies of the absolute dose and the irradiation localization among the participating institutions of the multicenter clinical trial were assured, and the JCOG clinical trials could be initiated.

## INTRODUCTION

In recent years, radiotherapy has become highly accurate owing to rapid technological progression. High-precision radiotherapy techniques, such as stereotactic body radiotherapy (SBRT), intensity-modulated radiotherapy (IMRT), volumetric-modulated arc therapy (VMAT), image-guided radiotherapy (IGRT), 4D radiotherapy and particle beam therapy, are capable of delivering with increased dose concentration to tumors and dose reduction to normal tissue. Therefore, high-precision radiotherapy is expected to improve the cure rate and reduce adverse events.

The Radiation Therapy Study Group (RTSG) in the Japan Clinical Oncology Group (JCOG) [1,2] has initiated several multicenter clinical trials using the aforementioned high-precision radiation therapies, and several new clinical trials are ongoing [3–10]. It is necessary to unify various physical quantities such as doses to targets and organs at risk and irradiation localization accuracy to conduct multi-center clinical trials. Standardization of physical quantities used by institutions directly improves the reliability of final clinical trial results. Thus, medical-physics-related verification of accuracy is essential [11]. Some countries have established their own medical-physics-related verification system for the absolute dose and irradiation localization and the support of radiotherapy multicenter clinical trials. The European Organisation for Research and Treatment of Cancer (EORTC) [12], Imaging and Radiation Oncology Core (IROC) Houston Quality Assurance Center [13], Radiotherapy Trials Quality Assurance (RTTQA) [14], Trans Tasman Radiation Oncology Group (TROG) Cancer Research [15], Australian Clinical Dosimetry Service (ACDS) [16], etc. are examples of groups that have adopted their own the medical-physics-related verification system. However, the medical-physics-related verification system is not adequately established to support radiotherapy multicenter clinical trials in Japan.

In 2011, the Medical Physics Working Group (MPWG) of the JCOG-RTSG (the first medical-physics-related verification system in Japan) was established to support the JCOG multicenter clinical trials using radiotherapy. Further, we performed credentialing of participating institutions in various JCOG multicenter clinical trials [17–26]. In this report, we introduce the verification method and the audit method established to examine the accuracy of the absolute doses and the image-guided irradiation localizations in SBRT, IMRT/VMAT and proton beam therapy (PT).

## Construction and activity of JCOG–RTSG–MPWG

The largest clinical research group in Japan, JCOG, was established in 1990. It currently consists of 16 research groups, one of which is the JCOG-RTSG. Five radiotherapy multicenter clinical trials have been initiated by the JCOG-RTSG, and four other clinical trials are in progress [27].

**Fig. 1.**
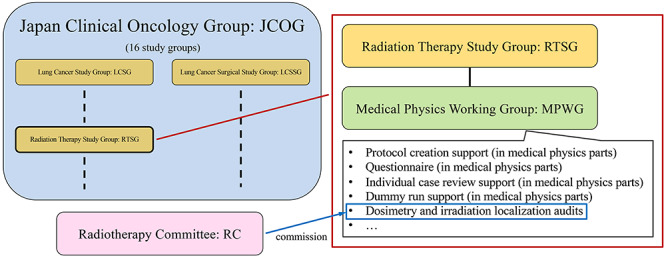
Correlation of the JCOG, the JCOG-RC and the JCOG-RTSG-MPWG, and a list of the medical physics QA supports.

Since 2003, few medical physicists have performed activities to support the quality assurance (QA) of physical quantities. JCOG0403, the first clinical trial in the JCOG-RTSG, started the registration of cases in July 2004. In 2011, under the JCOG-RTSG, the JCOG-RTSG-MPWG was officially launched, which consisted of expert medical physicists at each of Japan’s leading institutions [28]. Currently, 20 medical physicists are regular members of the JCOG-RTSG-MPWG. At the JCOG-RTSG-MPWG, we will support the creation of medical physics-related clinical trial protocols. For credentialing each clinical trial, we will conduct a questionnaire survey on technologies, such as the irradiation method of participating institutions, the performing of dummy runs, planning and implementation of visiting and postal audits for accuracy verification of absolute dose and irradiation localization. The commission of Radiotherapy Committee (RC) in the JCOG performs the credentialing. Correlation of the JCOG, the JCOG-RC and the JCOG-RTSG-MPWG and a list of the medical physics QA supports are shown in Fig. 1. Table 1 presents a list of the JCOG-RTSG multicenter clinical trials and the JCOG-RTSG-MPWG contributions. We have provided support to 243 clinical trial participants in nine JCOG radiotherapy multicenter clinical trials. Furthermore, we are also conducting international joint research in collaboration with the global QA of radiation therapy clinical trials harmonization group (GHG), which is described later in this report [29, 30, 31].

**Table 1:** List of JCOG-RTSG multicenter clinical trials and JCOG-RTSG-MPWG contributions

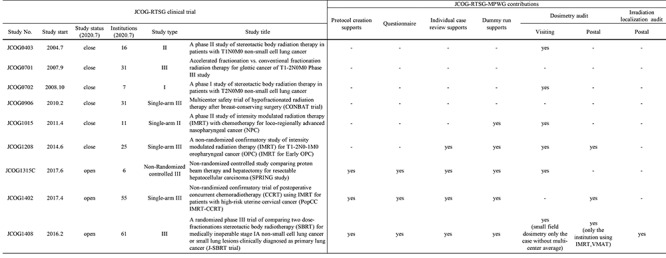

## Dosimetry and imaging audit system for lung-SBRT clinical trials: JCOG0403, 0702 and 1408

In lung SBRT, a high dose irradiation is delivered to the target in 5–12 Gy and 4–10 fractions resulting in 5–12 Gy per day. Furthermore, the difference between the target and the lung densities is large and heterogeneous on computed tomography (CT) images. Therefore, the dose calculation accuracy required in the treatment planning is high, and it is very important to assure the dose calculation accuracy. To date, lung SBRT multicenter clinical trials JCOG0403 [3] and JCOG0702 [5,6,7] have been initiated, and JCOG1408 (a joint study with the JCOG Lung Cancer Study Group) [10] is currently in operation.

The lung SBRT multicenter clinical trial JCOG0403 has been performing dose verification using a solid body phantom with a 3-cm-diameter simulated spherical tumor and a simulated lung, made from Toughwater (water equivalent material; Kyoto Kagaku Co. Ltd., Kyoto, Japan; physical density: 1.017 g/cm^3^; electron density: 3.25$\times$10^23^/g; elementary composition: 8.4% H, 67.4% C, 2.2% N, 19.5% O, 0.2% Cl, 2.3% Ca) and Toughlung (lung equivalent material; Kyoto Kagaku Co. Ltd., Kyoto, Japan; physical density: 0.32 g/cm^3^; electron density: 3.26$\times$10^23^/g; elementary composition: 7.0% H, 50.2% C, 35.1% O, 1.5% Al, 5.0% Si, 0.1% P, 1.0% Cl), respectively [17]. A radiophotoluminescent glass dosimeter, DOSE ACE (Asahi Glass Co., Tokyo, Japan), can be inserted in the center of the simulated tumor. In addition, it is possible to measure the dose distribution using an EDR2 film (Kodak Inc., New York, USA) on the axial plane that passes through the center of the simulated tumor.

**Fig. 2.**
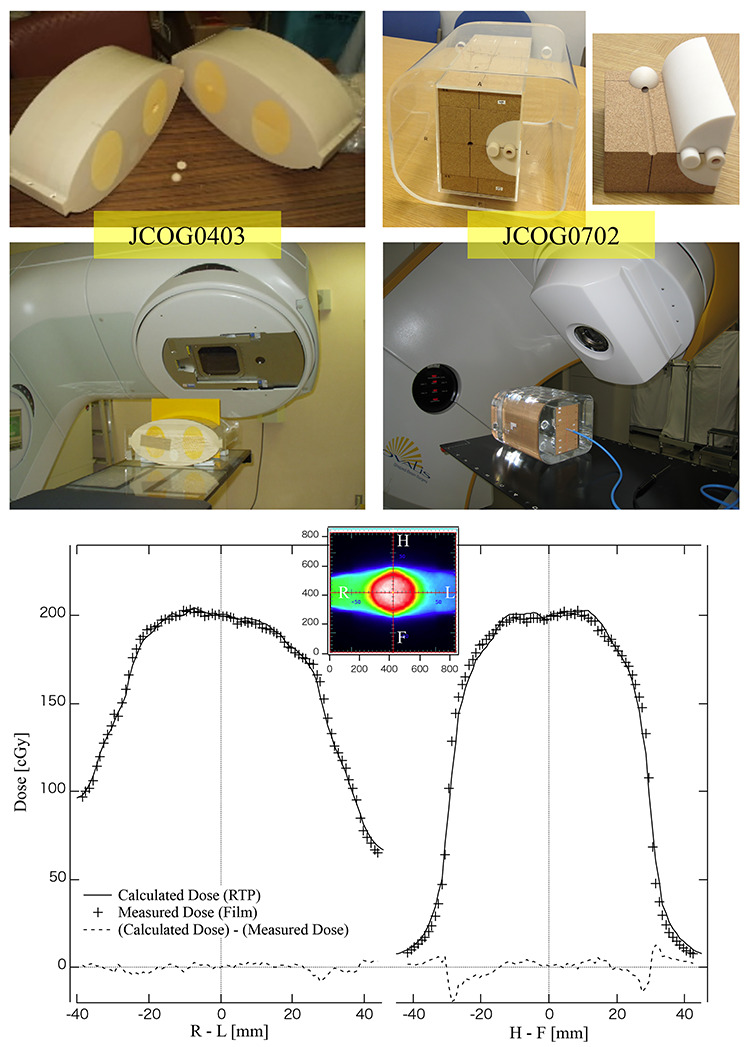
Lung phantom for the lung SBRT dosimetry audit system (upper-left: JCOG0403 phantom, upper-right: JCOG0702 phantom) and an example of the calculated and measured dose distributions in lung SBRT (lower).

The JCOG0702 multicenter clinical trial newly designed a water-tank-type lung phantom (Taisei Medical Inc., Osaka, Japan) [18]. It is possible to improve solid phantom-dependent dose calculation accuracy and reduce the weight of the phantom due to the solid phantom being replaced with the water-type phantom. The simulated lung and the mediastinum of this phantom were made of cork and Toughwater, respectively. A small volume-ionization chamber dosimeter, i.e. a PinPoint 3D air-filled ionization chamber 31016 (PTW, Freiburg, Germany), can be inserted at the center of a 3-cm-diameter simulated spherical tumor made of Toughwater, and the irradiation dose can be measured. The measurement of the dose distribution on the axial plane that passes through the center of the simulated tumor is performed using Gafchromic EBT films (Ashland Specialty Ingredients, New Jersey, USA). Using these tools, we performed an end-to-end test simulating actual treatment via CT imaging, treatment planning, dose calculation, phantom irradiation and dosimetry. By analyzing and comparing the dose measurement data and the dose calculation results, the accuracy of the presecription dose at each participating institution conducting the clinical trial was verified. Figure 2 shows the dosimetry audit phantom designed for lung SBRT and an example of dose distribution in lung SBRT. The results of dose verification by visiting dosimetry audit, in the JCOG0403 multicenter clinical trial, showed that the difference between the calculated dose and the measured dose in the center of the simulated tumor was within ±4% at all 16 clinical trial participating facilities [17]. In all seven JCOG0702 multicenter clinical trial participating facilities, the difference between the calculated and the measured doses at the center of the simulated tumor was within ±2% for all 30 irradiation plans and within ±4% for all 131 irradiation fields [18]. Dosimetry verification has been performed by the visiting audit thus far. We are currently developing a postal dosimetry audit system by improving this water-tank-type lung phantom.

**Fig. 3.**
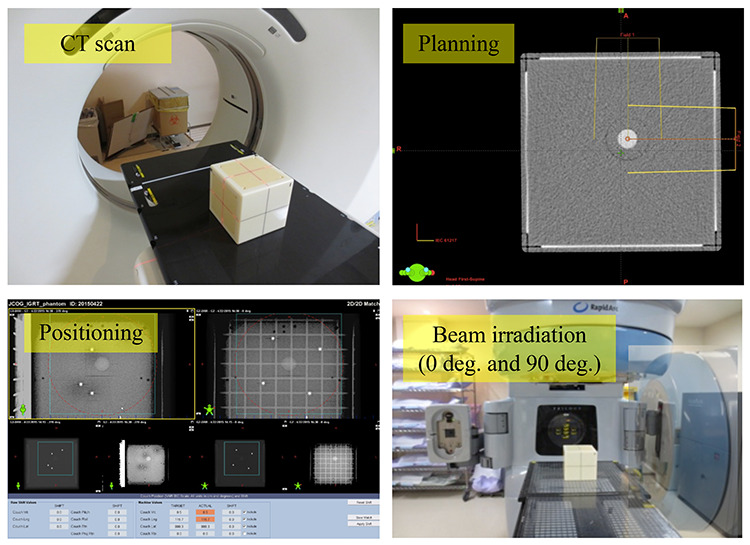
Procedure of the end-to-end test system for IGRT.

In the JCOG1408 multicenter clinical trial, validation for determining the prescription dose in the planning target volume (PTV) coverage [21], SBRT questionnaires of physical aspects including small field dosimetry check, comparison with multicenter average and respiratory motion management, and verification of the accuracy of image-guided irradiation localization were performed [22]. A 15 cm cube phantom for accuracy verification of image-guided irradiation localization (Taisei Medical Inc., Osaka, Japan) was designed as shown in Fig. 3. A Gafchromic RTQA2 film (Ashland Specialty Ingredients, New Jersey, USA), four gold fiducial markers of 1.5-mm diameter, and a spherical dummy target for confirming the irradiation localization of therapeutic X-rays were installed in this cube phantom. Thereby, the 3D coincidence between the irradiation localization of the therapeutic X-ray and the target position of the patient could be verified. A postal audit of the end-to-end test using this cube phantom was conducted at each JCOG1408 clinical-trial participating institution, and the coincidence of the irradiation localization of the therapeutic X-ray on the 6-axes was confirmed.

## Dosimetry audit system for IMRT/VMAT clinical trials: JCOG1015, 1208 and 1402

IMRT/VMAT will be utilized in various JCOG clinical trials not only in the JCOG-RTSG but also in other JCOG study groups using radiotherapy. The multicenter clinical trials that are representative of the JCOG-RTSG are JCOG1015 [9], JCOG1208 for head and neck, and JCOG1402 (a joint study with the JCOG Gynecologic Cancer Study Group) for the uterus and cervix. In JCOG1008, JCOG1212, JCOG1303, JCOG1703 and JCOG1910, which are representative of the JCOG Head and Neck Cancer Study Group and Brain Tumor Study Group, IMRT/VMAT is used.

**Fig. 4.**
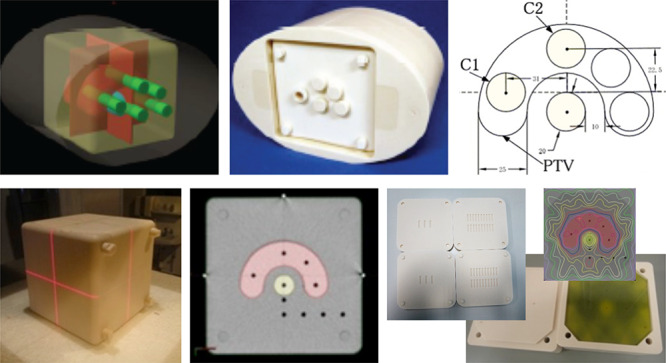
Phantom for the IMRT/VMAT dosimetry audit system (upper: visiting audit phantom, lower: postal audit phantom).

We designed an IMRT/VMAT dose verification phantom made of Toughwater for a visiting audit to ensure consistency of the absolute dose among the clinical-trial participating institutions (Fig. 4, upper). The IMRT/VMAT dose verification phantom consists of a base unit and a module unit. The dosimetry module for the air-filled Farmer ionization chamber and the Gafchromic EBT2 film (Ashland Specialty Ingredients, New Jersey, USA), and the CT imaging module are available in the dose verification phantom. Inside the CT imaging module is a cylindrical organ at risk (OAR) phantom and a horseshoe-shaped target phantom (TM Phantom; Taisei Medical Inc., Osaka, Japan; physical density: 1.000 g/cm^3^; electron density: 3.37$\times$10^23^/g; elementary composition: 11.6% H, 88.4% C) surrounding the OAR. Since the above-mentioned substances have a slightly lower density than that of Toughwater, their shapes are clearly outlined on CT images. A visiting dosimetry audit using an air-filled ionization chamber and dosimetric film was performed to verify and evaluate the absolute dose and the dose distribution at each institution [20]. The IMRT/VMAT treatment plan with a prescription dose of 2 Gy in 95% coverage of PTV and the maximum dose of PTV <110%, was performed for the dosimetry audit. The difference between the dose calculated in treatment planning systems and the dose measured by the air-filled ionization chamber was within ±3% at all clinical trial participating institutions. The calculated and measured dose distributions were evaluated on whether the positional differences of dose distribution at the 60 and 80% dose points was within ±2 mm.

**Fig. 5.**
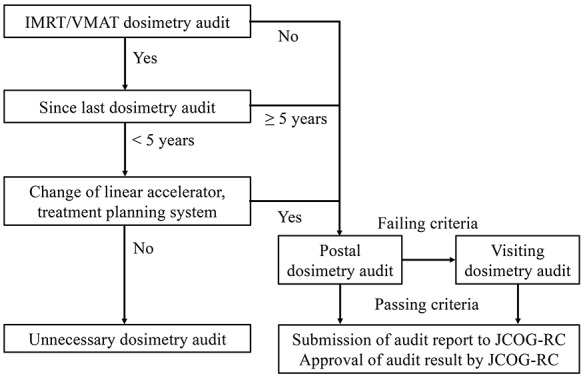
Logic of the IMRT/VMAT dosimetry audit system.

We modified the phantom design to change from a visiting audit to a postal audit (Fig. 4, lower). Subsequently, it was confirmed that the postal dosimetry audit could perform dose evaluation with the same accuracy as the visiting dosimetry audit [24]. In the IMRT/VMAT postal dosimetry audit, a radiophotoluminescent glass dosimeter is used to measure the absolute dose [32]. Currently, all IMRT/VMAT dose verification is conducted through postal dosimetry audit. The logic of the IMRT/VMAT dosimetry system is shown in Fig. 5. For evaluation of the dose distribution, we are currently using commercial software; however, the evaluation process is in the black-box. In an effort to eliminate the black-box and implement a new statistical-based algorithm for the dose distribution evaluation, we are attempting to develop in-house software.

## Dosimetry audit system for a liver proton therapy clinical trial: JCOG1315C

**Fig. 6.**
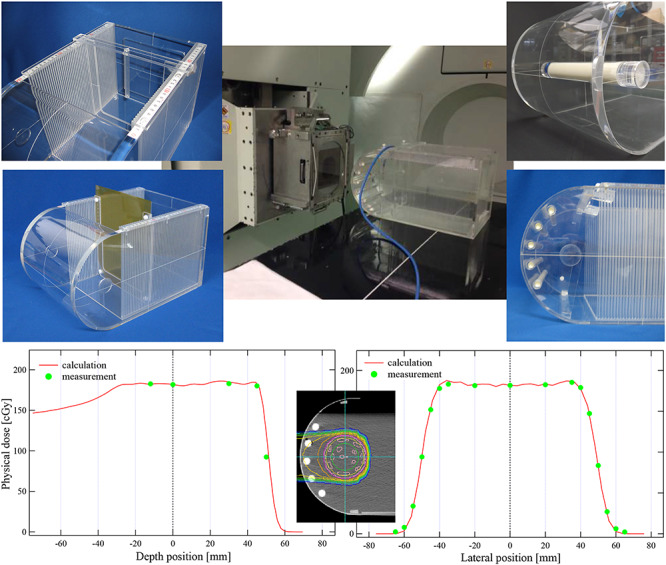
Liver and prostate phantom for the proton therapy dosimetry audit system (upper) and example of calculated dose distribution and measured point dose in the JCOG1315C dosimetry audit (lower).

The first domestic clinical trial involving PT for liver in Japan was initiated as the JCOG1315C multicenter clinical trial. The liver and prostate phantom (Taisei Medical Inc., Osaka, Japan) was designed to perform dosimetry verification in the JCOG1315C multicenter clinical trial (Fig. 6, upper) [25]. The ribs sub-phantom, made of BE-H Toughbone (bone equivalent material; Kyoto Kagaku Co. Ltd., Kyoto, Japan; physical density: 1.50 g/cm^3^; electron density: 3.16$\times$10^23^/g; elementary composition: 5.1% H, 42.5% C, 1.7% N, 28.1% O, 7.0% P, 0.1% Cl, 15.5% Ca), used in the dosimetry verification of the liver PT, and the femoral-head sub-phantom, made of BE-H, used in the dosimetry verification of the prostate PT, are installed in the water-tank-type base phantom. By holding the tools of the air-filled ionization chamber and the dosimetric film in the base phantom, it is possible to measure the absolute dose at any point and the dose distribution on any plane. The shape of the proton dose distribution is dependent on the manufacturer of each institution’s PT system. Moreover, passive beam and pencil beam scanning irradiation methods are used for the clinical proton dose configuration in PT. Therefore, providing QA of the proton dose irradiation to a target among participating institutions is challenging.

On performing the visiting dosimetry audit, the difference between the calculated and the measured doses was observed to be within ±3% at all six institutions participating in the clinical trial, and the JCOG1315C multicenter clinical trial with all institutions could be initiated (Fig. 6, lower).

## International collaboration for ensuring QA of the physical quantities

As mentioned above, QA in clinical trials is crucial; a lack of compliance can affect the trial outcome [11, 33–36]. Different international radiotherapy clinical-trial QA groups have developed independent methods of measuring dose distribution verification and analysis for various historical and logistical purposes, with the ultimate aim of ensuring compliance [37–40].

Individual clinical-trial QA groups have streamlined methods for multiple trials to avoid duplication. Some clinical trials are now open to international recruitment to increase patient numbers and limit the time to full accrual. Streamlining dosimetry QA in the international setting, such that an institution credentialed by one QA group may be accepted by another, is therefore of interest. To be able to achieve this, it is important to understand how different analysis techniques and tolerances translate between different groups, and the challenges involved therein. The GHG has been established to facilitate harmonization and reciprocity of clinical trial QA between different groups and consistency in dose delivery in the trials [29, 41–43].

The mission of GHG is to promote harmonization of radiotherapy QA between trial groups globally.

The goals and strategy of GHG are as follows. (i) Bring together, harmonize and distribute information regarding the QA of radiation therapy (RTQA) standards of various trial groups in clinical trials. (ii) Provide a platform for prospective discussions on new RTQA levels, software tools, guidelines and policies of trial groups. (iii) Provide a framework to endorse existing and future RTQA levels and guidelines between various trial groups. Each organization will be able to specify which RTQA procedures from other organizations they endorse and thus accept for future collaborative trials. (iv) Promote high-quality RTQA for clinical trials.

JCOG-RTSG-MPWG is actively engaged in GHG.

## Future work and visions

The JCOG-RTSG-MPWG performed credentialing of the absolute dose and the irradiation localization for the participating institutions in the JCOG multicenter clinical trial, and provided support for the protocol preparation and dummy run for each clinical trial. We have developed verification tools and systems suitable for the JCOG multicenter clinical trials using various treatment methods and irradiation techniques, such as SBRT, IMRT/VMAT and PT. Furthermore, we have developed a postal auditing system that responds to the increasing number of institutions who participate in clinical trials. As a result, accurate standardization of the absolute dose and the irradiation localization among the clinical trial participating institutions was assured, and JCOG clinical trials of high quality could be initiated. Conversely, radiation therapy technology continues to develop every day. Therefore, it is in our best interest to develop new verification methods corresponding to various high-precision radiotherapy methods, such as 4D radiotherapy and treatment methods using artificial intelligence, and to establish and maintain the QA support system.

In the future, apart from standardization of the new radiotherapy method used by the JCOG-RTSG, the JCOG-RTSG-MPWG will need to aim for the standardization of medical-physics-related verification in various radiotherapies. It will be necessary to establish a medical-physical-related verification system that can perform QA assessment of radiotherapy not only in clinical trials but also in institutions, including designated cancer hospitals. It will also be significant for the JCOG-RTSG-MPWG to standardize the QA of radiotherapy in Japan globally through international collaboration with medical-physics-related verification groups in other countries.
